# Seven-State Study of Assisted Living and Healthcare Providers’ Responses to COVID-19

**DOI:** 10.1093/geroni/igab046.220

**Published:** 2021-12-17

**Authors:** Sheryl Zimmerman, Philip Sloane, Johanna Hickey, Christopher Wretman, Paula Carder, Kali Thomas

**Affiliations:** 1 Cecil G. Sheps Center for Health Services Research, Chapel Hill, North Carolina, United States; 2 UNC Medical School, Sheps Center, Chapel Hill, North Carolina, United States; 3 University of North Carolina at Chapel Hill School, Chapel Hill, North Carolina, United States; 4 OHSU-PSU School of Public Health, Portland, Oregon, United States; 5 Brown University, Brown University/Providence, Rhode Island, United States

## Abstract

COVID-19 has inordinately affected assisted living (AL), such that the proportion of fatalities to cases has been 21% in AL versus 2.5% for the general population. Understanding how AL administrators and medical and mental health providers have responded to COVID-19 can inform health care going forward. Using a seven-state stratified random sample of 250 communities, administrators were interviewed and providers completed questionnaires regarding COVID-19 practices. Preliminary data indicate that 79%, 44%, and 62% of administrators reported serving meals in rooms to segregate residents, using telemedicine, and providing extra pay for staff, respectively. Perceived use/effectiveness of practices differed based on dementia case-mix (e.g., face coverings, social distancing). Providers reported less access to patients (82%), more telehealth (63%), and less ability to provide care (43%). However, they uniformly reported high confidence in AL staff ability to prevent (94%) and respond to outbreaks (96%). Discussion will summarize points important for future care.

